# The Discovery of Chloramphenicol Treatment for Both Scrub Typhus and Typhoid Fever

**Published:** 2025-04-20

**Authors:** G. Dennis Shanks

Today, the U.S. and its allies collaborate on missions throughout the globe, able to deploy in tropical regions without the massive disease casualties of 20th century conflicts. During World War II, at the dawn of the antibiotic era, thousands of Allied soldiers in the Pacific died of an untreatable illness, *tsutsugamushi* , or scrub typhus, a rickettsial infection endemic to Southeast Asia. An additional tens of thousands suffered non-fatal infections, often incapacitated for months. ^1^

When the U.S. Typhus Commission was formed in 1942, its focus was epidemic typhus in Europe, but it came to include scrub typhus in the Pacific. Research to find an effective treatment for scrub typhus was a military priority, and Dr. Joseph Smadel, Chief of the Department of Virus and Rickettsial Diseases at the Walter Reed Army Institute of Research (WRAIR), was focused on these efforts.

Trials initiated by Dr. Smadel in partnership with scientists in the then-British colony of Malay, now Malaysia, resulted in the serendipitous discovery of treatment for 2 major infectious diseases. The U.S. Army Medical Research Unit–Malaysia resulted from initially informal collaborations between Dr. Smadel and WRAIR researchers and British scientists at the Institute for Medical Research in Kuala Lumpur.

Within a year of chloramphenicol's discovery in 1947, Dr. Smadel had collected most of the existing stock—it would not be fully synthesized until 1949—for field trials in Malaysia. Smadel first tested the drug in rickettsial laboratory cultures and then progressed to field trials in naturally infected rubber plantation workers in Malaysia. ^2^

Within 6 weeks, in early 1948, 25 scrub typhus patients had been successfully treated with chloramphenicol. Patient fevers cleared in an average of 31 hours, despite total treatment duration as brief as a single day. This victory against disease was considered noteworthy enough to warrant an editorial cartoon, printed in a Malaysian English language newspaper, evoking the U.S. Marines on Iwo Jima **(Figure)** .

**FIGURE.**
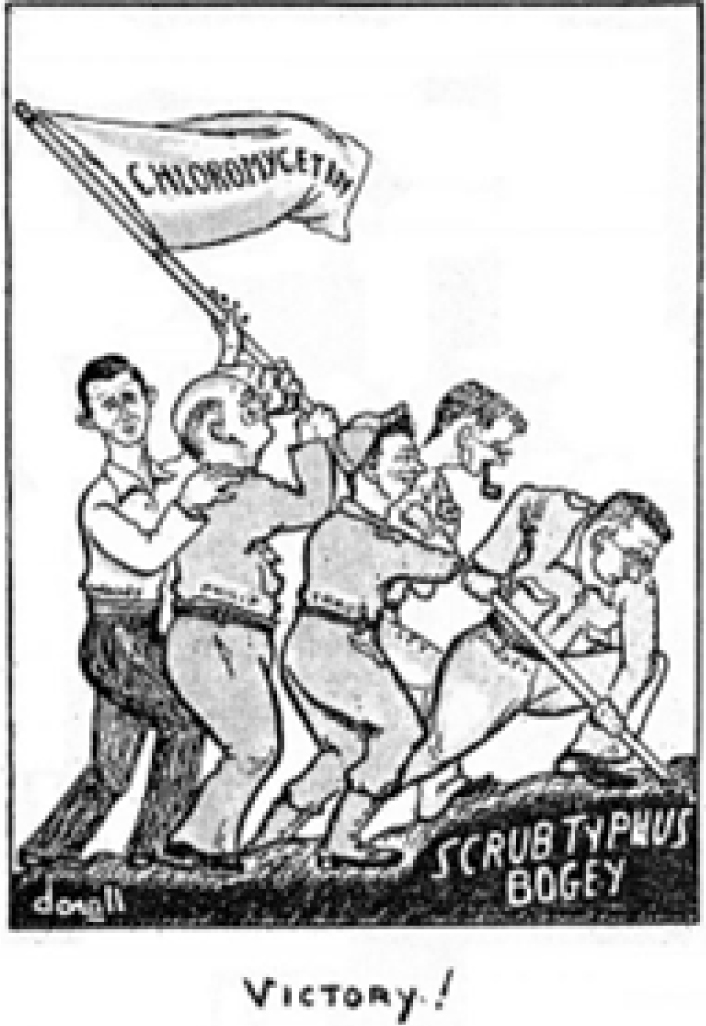
Cartoon in *The Malay Post* , ca. 1948, of the Joint Civil-Military Medical Team that Discovered Chloramphenicol Treatment for Lethal Rickettsial Infection of Scrub Typhus

Inadvertently, some initially mis-diagnosed typhoid fever patients were treated along with scrub typhus patients, and were found to be cured equally well. Ten typhoid cases received chloramphenicol, with fever clearance in 3.5 days; only 2 relapsed within 16 days, but subsequently responded well to re-treatment. ^3^

In only a few months, definitive treatments to 2 lethal, infectious diseases had been discovered by clinical trials by a U.S. Army medical research team from WRAIR, working collaboratively with local partners. These dual achievements were recognized in 1962 by the Lasker Clinical Award, which was awarded to Dr. Smadel.

The scale and speed of the discovery of scrub typhus and typhoid treatment were unique, but important later discoveries were made at other WRAIR laboratories. Since World War II, WRAIR has operated more than a dozen laboratories overseas. Japanese encephalitis and hepatitis A vaccines were field-tested at the Armed Forces Research Institute of Medical Sciences (AFRIMS) in Thailand, and mefloquine and tafenoquine were tested for malaria at AFRIMS and U.S. Army Medical Research Unit-Kenya. ^4 , 5^ With often no perceived pharmaceutical profit potential in Western nations for new treatments for exotic diseases, the research and development by WRAIR laboratories and their partners are of even greater importance.

The author, of both *Images in Health Surveillance* featured in this issue, acknowledges the service and sacrifice of all those who served in the military during the Second World War and thanks the many unnamed military officers, scientists, historians, and medical librarians who have unselfishly provided data and ideas for these manuscripts, especially the librarians at the Australian Defence Force Library at Gallipoli Barracks, Queensland.
